# Supplementary material for: Improvement of the Surface Properties of Polyether Ether Ketone via Arc Evaporation for Biomedical Applications
Source: Materials (Basel). 2023 Apr 9;16(8):2990. doi: 10.3390/ma16082990 (PMC10145034; doi:10.3390/ma16082990)
Supplement: Supplementary file 1 [file materials-16-02990-s001.zip › materials-2282407-supplementary.pdf]

Support Information of: Improvement of the Surface Properties of Polyether Ether Ketone via Arc Evaporation for Biomedical Applications

Alexander Y. Fedotkin [1,†], Igor O. Akimchenko [1,†], Tuan-Hoang Tran [1], Artur R. Shugurov [1,2], Evgeniy V. Shesterikov [3], Anna I. Kozelskaya [1], Sven Rutkowski [1,*] and Sergei I. Tverdokhlebov [1,*]

[1] Weinberg Research Center, School of Nuclear Science & Engineering, Tomsk Polytechnic University, 30, Lenin Avenue, 634050 Tomsk, Russia
[2] Institute of Strength Physics and Materials Science, Siberian Branch of the Russian Academy of Sciences, 2/4, pr. Akademicheskii, 634055 Tomsk, Russia
[3] Nanotechnology Research and Education Center, Tomsk State University of Control Systems and Radioelectronics, 40, Lenin Avenue, 634050 Tomsk, Russia
* Correspondence: rutkowski_s@tpu.ru (S.R.); tverd@tpu.ru (S.I.T.)
† These authors contributed equally to this work.

Supporting Figures:

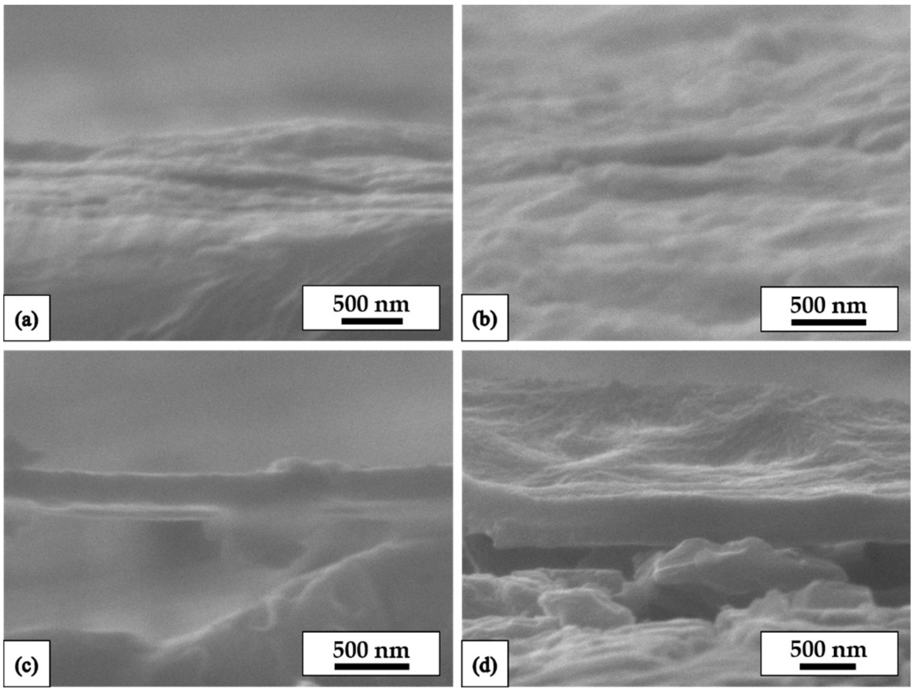

Figure S1. Cross sectional SEM micrographs of the surface-modified 3D–PEEK samples: (a) after 1 minute, (b) after 3 minutes, (c) after 5 minutes and (d) after 10 minutes of cathodic arc evaporation.

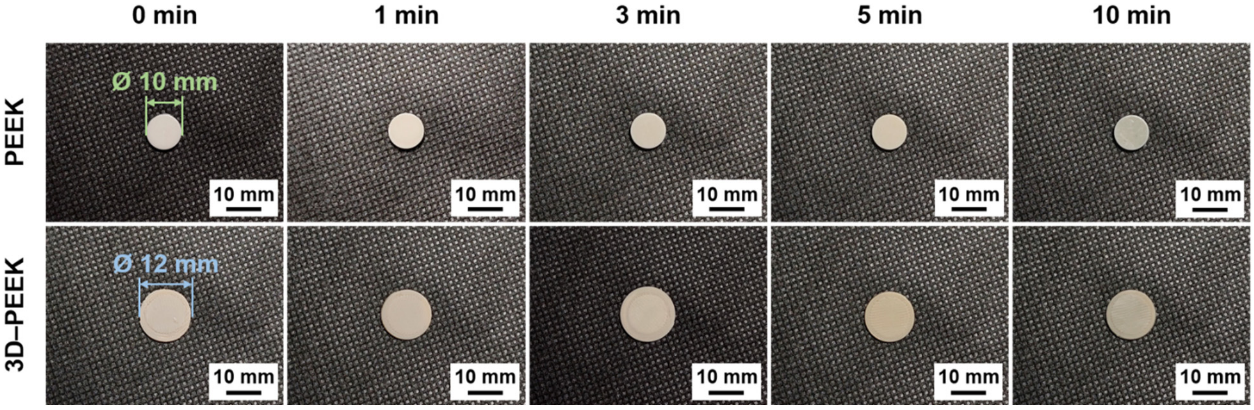

Figure S2. Photographs of the macroscopic appearance of the polished PEEK samples cut from extruded polymer bar (polished PEEK) and 3D-printed PEEK (3D–PEEK) samples before (0 min) and after modification by cathodic arc evaporation after 1 min, 2 min, 3 min, 5 min, and 10 min.

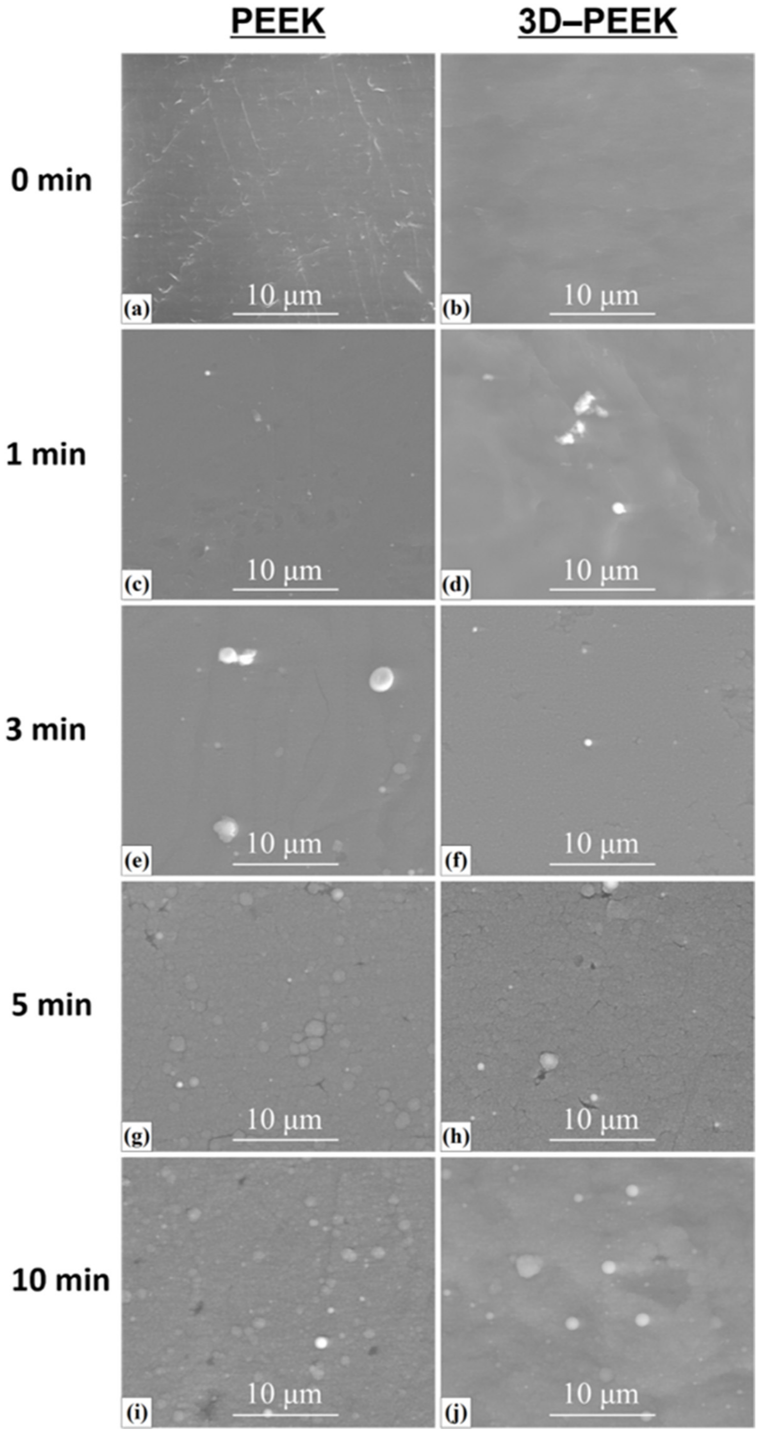

Figure S3. SEM images of the samples under study with arc evaporator processing time: unmodified polished PEEK sample (a), unmodified 3D–PEEK sample (b), PEEK–1 sample (c), 3D–PEEK–1 sample (d), PEEK–3 sample (e), 3D–PEEK–3 sample (f), PEEK–5 sample (g), 3D–PEEK–5 sample (h), PEEK–10 sample (i), 3D–PEEK–10 sample (j).

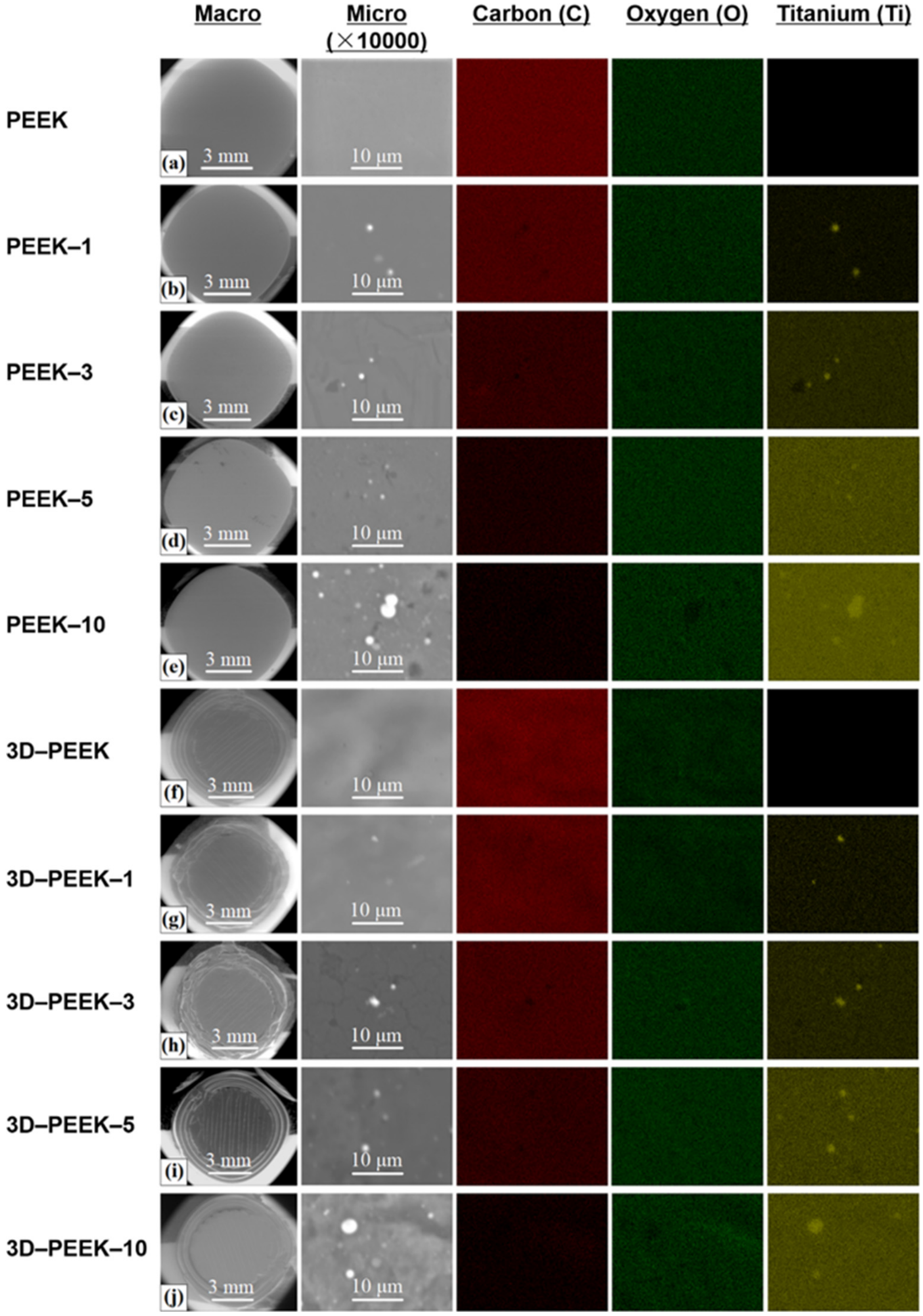

Figure S4. EDX mapping micrographs of the elemental composition of the studied samples for the elements carbon (C), ogyxen (O) and titanium (Ti): unmodified PEEK sample (**a**), PEEK–1 sample (**b**), PEEK–3 sample (**c**), PEEK–5 sample (**d**), PEEK–10 sample (**e**), unmodified 3D–PEEK sample (**f**), 3D–PEEK–1 sample (**g**), 3D–PEEK–3 sample (**h**), 3D–PEEK–5 sample (**i**), 3D–PEEK–10 sample (**j**).

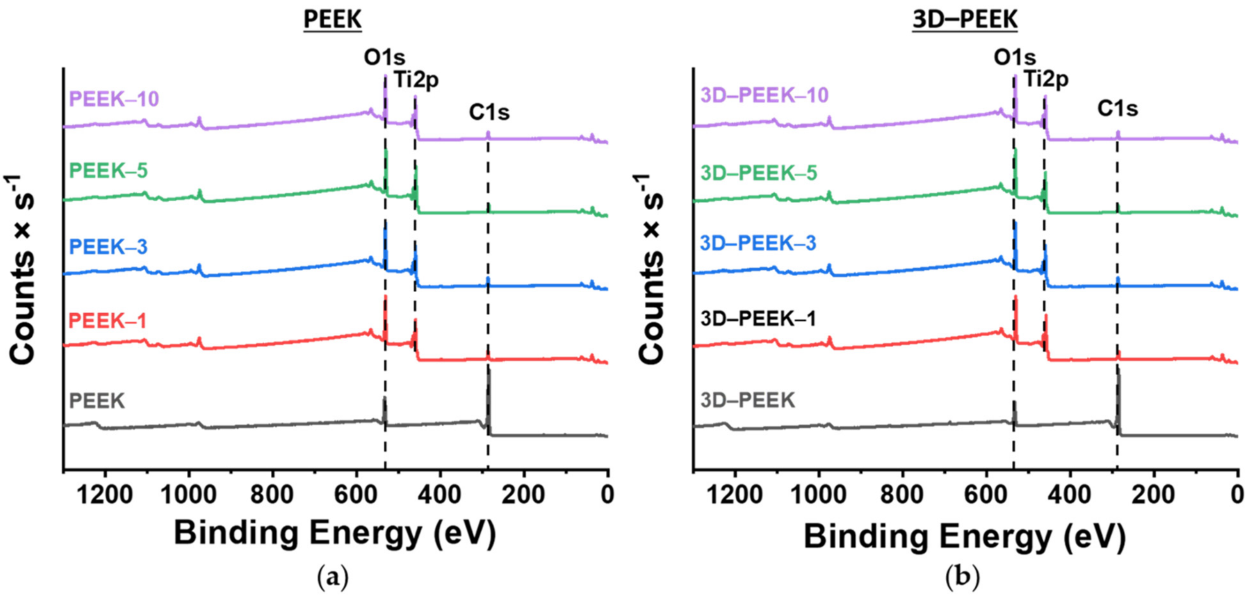

Figure S5. XPS survey spectra of all investigated PEEK samples.

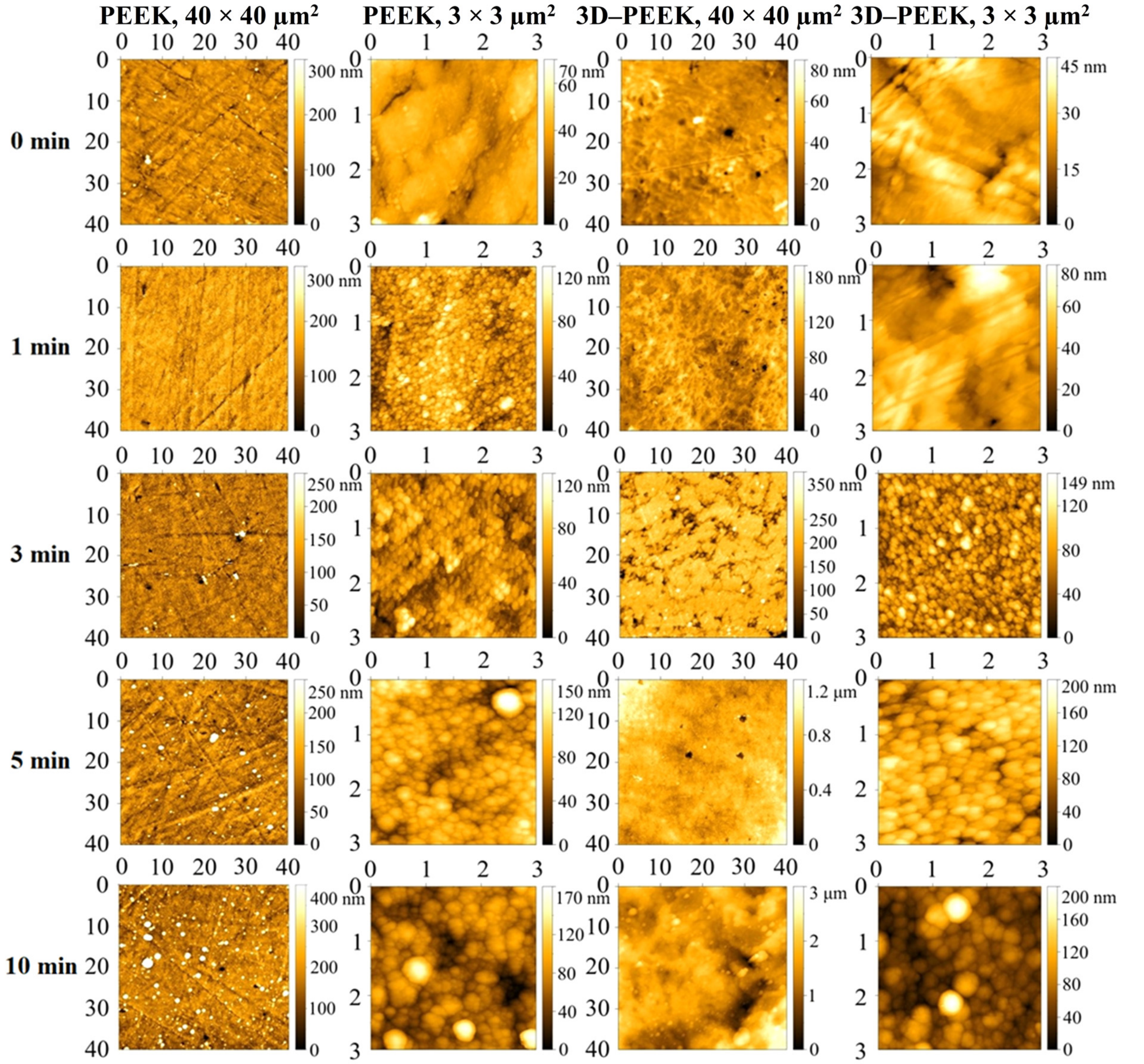

Figure S6. AFM images of the studied coatings with an area of 40 × 40 μm² (left) and 3 × 3 μm² (right): unmodified polished PEEK sample, PEEK–1 sample, PEEK–3 sample, PEEK–5 sample, PEEK–10 sample, unmodified 3D–PEEK sample, 3D–PEEK–1 sample, 3D–PEEK–3 sample, 3D–PEEK–5 sample, 3D–PEEK–10.

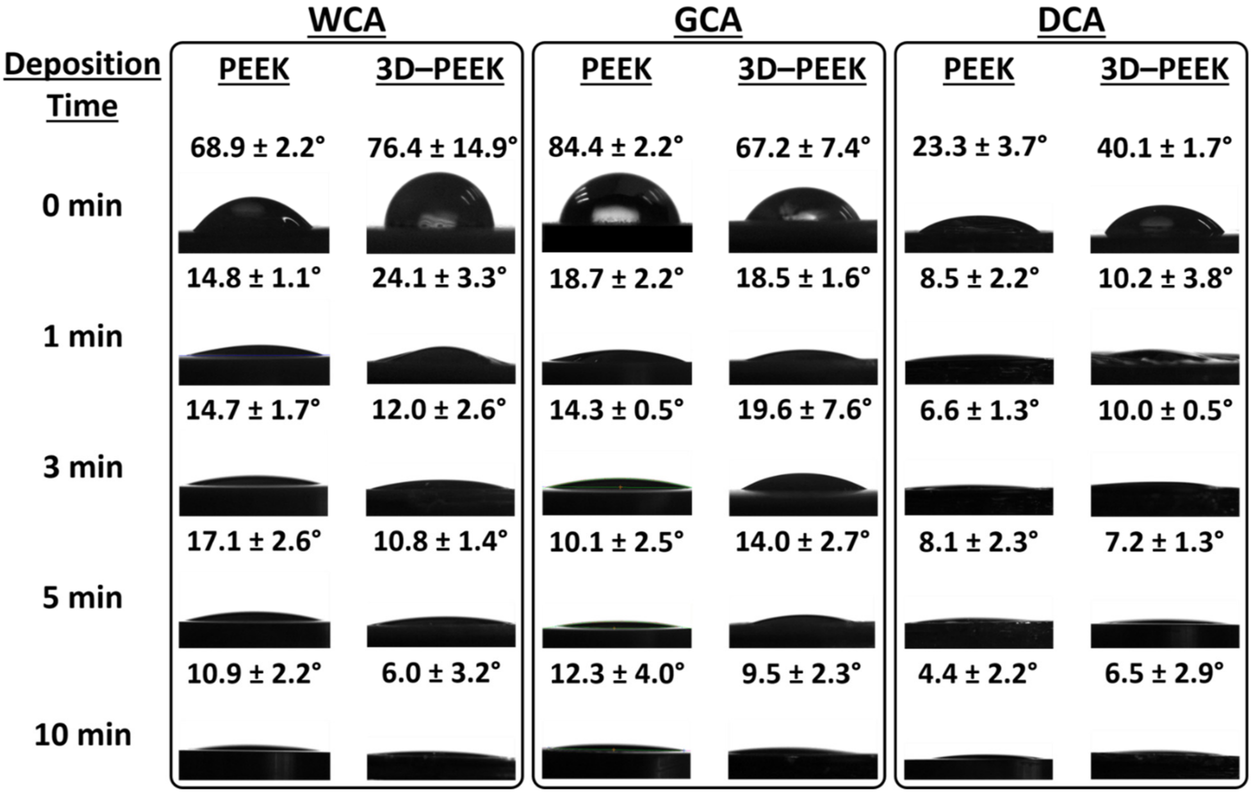

Figure S7. Micrographs of water, glycerol and diiodomethane droplets on the surfaces of each sample investigated with the indication of the measured values for the water contact angles (WCA), glycerol contact angles (GCA) and diiodomethane contact angles (DCA).

Supporting Tables:

Table S1. Elemental composition of all investigated PEEK samples for the elements carbon (C), oxygen (O) and titanium (Ti), as well as the calculated grade of O bound to C, the grade of O bound to Ti and the ratio of Ti/O.

Sample	C (at.%)	O (at.%)	Ti (at.%)	O bound to C (at.%)	O bound to Ti (at.%)	Ti/O
PEEK	85.47 ± 0.10	14.53 ± 0.10	–	0.17	–	–
PEEK–1	81.99 ± 0.13	17.09 ± 0.11	0.92 ± 0.04	13.12	3.97	0.23
PEEK–3	63.19 ± 1.06	30.12 ± 0.75	6.69 ± 0.32	10.11	20.01	0.33
PEEK–5	39.06 ± 3.58	45.17 ± 2.03	15.76 ± 1.56	6.25	38.92	0.40
PEEK–10	15.83 ± 1.81	54.54 ± 0.77	29.63 ± 1.23	2.53	52.01	0.57
3D–PEEK	85.23 ± 0.14	14.77 ± 0.14	–	0.17	–	–
3D–PEEK–1	82.53 ± 0.45	16.85 ± 0.45	0.60 ± 0.02	14.30	2.55	0.24
3D–PEEK–3	71.42 ± 0.28	24.78 ± 0.21	3.80 ± 0.07	12.38	12.40	0.31
3D–PEEK–5	56.89 ± 1.18	34.70 ± 0.69	8.41 ± 0.54	9.86	24.84	0.34
3D–PEEK–10	16.45 ± 1.43	53.94 ± 0.71	29.61 ± 0.95	2.85	51.09	0.58

Table S2. Concentration of the elements carbon, oxygen and titanium determined by XPS. Values are given as mean ± standard deviation and in atomic percent (at.%).

Sample	C1s (at.%)	O1s (at.%)	Ti2p (at.%)
PEEK	84 ± 2	16 ± 1	–
PEEK–1	25 ± 2	49 ± 2	26 ± 2
PEEK–3	24 ± 2	49 ± 2	26 ± 3
PEEK–5	23 ± 2	50 ± 2	27 ± 2
PEEK–10	23 ± 1	51 ± 3	27 ± 2
3D–PEEK	86 ± 2	14 ± 1	–
3D–PEEK–1	27 ± 3	48 ± 2	24 ± 2
3D–PEEK–3	27 ± 3	49 ± 2	24 ± 2
3D–PEEK–5	25 ± 3	50 ± 2	25 ± 2
3D–PEEK–10	23 ± 1	50 ± 1	27 ± 1

Table S3. Microroughness values of all investigated PEEK samples obtained by AFM at different scanning areas.

Sample	R_a (40 × 40) (µm^2)	R_a (3 × 3) (µm^2)	R_z (40 × 40) (µm^2)	R_z (3 × 3) (µm^2)
PEEK	17.67 ± 0.65	8.48 ± 3.10	99.33 ± 6.09	25.3 ± 7.47
PEEK–1	21.00 ± 0.40	14.82 ± 1.52	125.12 ± 0.83	58.04 ± 5.27
PEEK–3	19.05 ± 2.40	11.77 ± 2.68	114.35 ± 15.06	42.88 ± 8.07
PEEK–5	21.43 ± 2.31	12.28 ± 3.32	132.35 ± 10.11	40.51 ± 7.60
PEEK–10	40.37 ± 1.99	15.21 ± 1.95	252.10 ± 13.77	46.35 ± 6.03
3D–PEEK	37.44 ± 6.51	5.95 ± 1.05	140.85 ± 34.58	16.33 ± 1.56
3D–PEEK–1	34.24 ± 1.30	13.63 ± 9.92	168.5 ± 15.68	44.76 ± 23.45
3D–PEEK–3	38.04 ± 7.86	14.13 ± 0.92	188.85 ± 36.42	58.47 ± 5.22
3D–PEEK–5	85.67 ± 7.46	20.66 ± 3.67	316.16 ± 29.76	70.67 ± 14.39
3D–PEEK–10	105.28 ± 19.97	22.29 ± 9.39	453.57 ± 79.96	66.75 ± 23.16

Table S4. Contact angles of the studied samples: water contact angles (WCA). glycerol contact angles (GCA) and diiodomethane contact angles (DCA). Surface engery (σ) of the samples. as well as the polar (σ_P) and the dispersion (σ_D) components.

Sample	WCA (°)	GCA (°)	DCA (°)	σ (mN/m)	σ_P (mN/m)	σ_D (mN/m)
PEEK	68.9 ± 2.2	84.4 ± 2.2	23.3 ± 3.7	32.1 ± 0.8	11.1 ± 0.3	20.9 ± 0.5
PEEK–1	14.8 ± 1.1	18.7 ± 2.2	8.5 ± 2.2	74.7 ± 0.8	31.6 ± 0.3	43.1 ± 0.5
PEEK–3	14.7 ± 1.7	14.3 ± 0.5	6.6 ± 1.3	64.7 ± 0.5	23.7 ± 0.2	41.0 ± 0.3
PEEK–5	17.1 ± 2.6	10.1 ± 2.5	8.1 ± 2.3	73.2 ± 0.9	27.3 ± 0.4	45.9 ± 0.5
PEEK–10	10.9 ± 2.2	12.3 ± 4.0	4.4 ± 2.2	77.8 ± 0.9	29.3 ± 0.4	48.4 ± 0.5
3D–PEEK	76.4 ± 14.9	67.2 ± 7.4	40.1 ± 1.7	42.0 ± 0.8	2.6 ± 0.4	39.4 ± 0.4
3D–PEEK–1	24.1 ± 3.3	18.5 ± 1.6	10.2 ± 3.8	64.5 ± 1.3	26.7 ± 0.6	37.8 ± 0.7
3D–PEEK–3	12.0 ± 2.6	19.6 ± 7.6	10.0 ± 0.5	79.1 ± 0.4	29.1 ± 0.3	50.0 ± 0.1
3D–PEEK–5	10.8 ± 1.4	14.0 ± 2.7	7.2 ± 1.3	78.1 ± 0.5	29.5 ± 0.2	48.6 ± 0.3
3D–PEEK–10	6.0 ± 3.2	9.5 ± 2.3	6.5 ± 2.9	71.9 ± 1.2	28.9 ± 0.5	42.9 ± 0.6

Table S5. Mechanical properties of the unmodified PEEK samples: P_m – maximum applied load. h_m – maximum penetration depth. H – hardness. E^* – reduced Young's modulus. R – elastic recovery.

Sample	P_m (mN)	h_m (nm)	H (GPa)	E^* (GPa)	R
PEEK	20.00	1699 ± 33	0.33 ± 0.02	5.80 ± 0.20	0.21
3D–PEEK	20.00	2174 ± 213	0.22 ± 0.04	3.40 ± 0.50	0.24
